# Prevalence of post-traumatic stress disorder and associated predictors among mothers of preterm infants in Western Kenya: a cross-sectional study

**DOI:** 10.11604/pamj.2023.44.194.37849

**Published:** 2023-04-20

**Authors:** Beatrice Mukabana, Drusilla Makworo, Catherine Syombua Mwenda

**Affiliations:** 1 School of Nursing, Midwifery and Paramedical Sciences, Masinde Muliro University of Science and Technology (MMUST), Kakamega, Kenya,; 2 School of Nursing, Jomo Kenyatta University of Agriculture and Technology (JKUAT), Juja, Kenya,; 3 School of Nursing, South Eastern Kenya University (SEKU), Kitui, Kenya

**Keywords:** Newborn unit, preterm infants, post-traumatic stress disorder, predictors

## Abstract

**Introduction:**

mothers of preterm infants are exposed to stress leading to post-traumatic stress disorder (PTSD). Preterm births have increased lately with World Health Organization (WHO) reporting an estimated prevalence of up to 5-18% and Kenya reporting a prevalence of 18.3%. The current study sought to determine the prevalence of PTSD and its associated predictors among mothers with preterm infants.

**Methods:**

this was a hospital-based cross-sectional study among 182 mothers with preterm babies admitted in neonatal care units (NCUs) of two referral hospitals. A simple random sampling technique was used to select participants and data was collected using a semi-structured pretested questionnaire and an Impact of events scale-revised (IES-R). Analysis was done using STATA 15 and a significance level set at P≤ 0.05 and 95% confidence interval.

**Results:**

the majority of the respondents 67 (36.8%) were aged between 18-22 years and only 34 (18.7%) were above 34 years. Most of the respondents had attained secondary and tertiary level education at 86 (47.3%) and 51 (28.0%) respectively. Prevalence of PTSD was 78.6% at a confidence interval of 95% CI: 0.72-0.84. Mothers who had a previous preterm birth were 0.09 less likely to develop PTSD {AOR=0.09, 95% CI: 0.01-0.80, p=0.023} while those who underwent cesarean section were 11.1 times more likely to develop PTSD {AOR=11.1, 95% CI:1.1-114.8, p=0.043}.

**Conclusion:**

the prevalence of PTSD was 78.6%. Although mothers of preterm infants experience stress, the associated predictors included; cesarean section birth, having had a preterm birth before and staying in fair housing conditions.

## Introduction

Preterm babies are those born before 37 complete weeks of gestation and because of their prematurity, they are admitted to the Neonatal care unit (NCU) also known as newborn care unit (NBU) where they receive specialized care [1]. They are categorized into three basing on their gestational period, that is, extremely preterm (less than 28 weeks gestational age), very preterm (28 - 32 weeks of gestational age) and moderate to late preterm (32- less than 37 weeks of gestational age) [2]. According to the National guidelines on quality obstetrics and perinatal care [2], extremely preterm and very preterm babies are usually unstable with many complications because their internal organs are immature. This calls for admission into specialized neonatal care unit unlike the moderate and late preterm who can be admitted in Kangaroo mother care rooms depending on their condition. The World Health Organization has estimated the prevalence of premature birth to be 5-18% across around 184 countries of the world and in Kenya, a prevalence of 18.3% has been noted in a study carried out at Kenyatta National Hospital (KNH) [3]. A study carried out at the Kakamega County Teaching and Referral Hospital also noted a prevalence of preterm births to be 18.6% [4]. United Nations International Children's Emergency Fund (UNICEF) [5] on the other hand noted that in Kenya, 12 out of 1000 live births are premature births. Usually, parents are unprepared for the unexpected early birth, making them face emotional problems [6]. According to Alsaiari *et al*. [1] premature babies have unstable vital signs and irregular temperatures. These, accompanied by the appearance of the infant, the behavior, the vulnerable state of the baby and the complex treatment technology predisposes the mothers to mental health problems. Premature birth and subsequent hospitalization of infants in NCU are highly traumatic and distressing for mothers and this puts them (mothers) at risk for the development of negative mental health outcomes like post-traumatic stress disorder, as well as other negative emotional outcomes like postnatal depression, and negative mood state [7-10].

During the stay in NCU, the adverse medical conditions of the baby plus other traumatic events like invasive procedures and life and death struggles of the infants prevent parents from immediately taking up their parental role. This may lead parents to experience higher levels of psychological distress because they have been denied an opportunity to take care of their infant [11,12]. A meta-analytic study on the global perspective on parental stress related to NCU admission concluded that parental stress associated with NCU admission is a worldwide healthcare problem that needs to be addressed [13]. Post-traumatic stress disorder (PTSD) is a mental disorder that may develop after exposure to exceptionally threatening or horrifying events, in this case, preterm birth. It can occur after a single traumatic event or from prolonged exposure and predicting who will go on to develop PTSD is a challenge [14]. Patients with PTSD presents with feelings of being detached from their own self and feels like the world seems unreal and dreamlike [15]. According to Courtois *et al*. [16], several guidelines from professional bodies have recommended trauma focused psychological interventions in managing PTSD though medical treatment can also be beneficial. Due to the negative effects of preterm birth on mothers´ emotional states, it is necessary to identify which parents are at risk by determining the predictors. Identifying the predictors could help to direct specific interventions that can reduce these parents´ psychological distress and prevent them from developing negative feelings [11-14,17]. Despite the high premature infants´ rates and the perceived impact these have on maternal emotional states, few studies have been conducted to determine the prevalence of PTSD and its associated predictors in low middle-income countries (LMIC) [18,19]. The purpose of the study was to determine the prevalence of PTSD and its associated predictors among mothers of preterm infants admitted in NCU as identifying the predictors could help the policymakers direct specific interventions that can reduce these mothers´ psychological distress and prevent them from developing negative feelings. The results from the study will help policymakers develop interventions that will guide mothers with babies in NCUs in order to prevent the risks for future PTSD.

## Methods

**Study design:** it was a hospital-based analytical cross-sectional study among mothers with preterm infants admitted in neonatal care unit and data collection was done from June through August 2021.

**Study population:** mothers with babies born before 37 weeks gestation admitted in NCU for at least five days or more. This was on assumption that at least five days or more was long enough for the mother to report how the situation had affected her.

**Setting:** Kakamega and Vihiga County Teaching and Referral Hospitals (KCTRH and VCTRH) located in the country’s western part. The two hospitals are the largest in Kakamega and Vihiga counties and have a bed capacity of 320 and 300 and register approximately 4% and 3% preterm births per month respectively. The District health information system (DHIS) (2018) report indicates that slightly above 90% of preterm infants end up in specialized neonatal care unit due to the accompanying baby complications including immature organs, with only approximately 10% of the preterm infants who are stable being admitted to Kangaroo mother care room (KMC) immediately after birth. Nevertheless, those admitted to KMC may still be admitted to NCU if their condition changes.

**Sample size and sampling technique:** sample size determination was based on Yamane´s Formula by Boore [20] which is used when the study population (N) is known, that is;


$$n=\frac{N}{1+N{\left(e\right)}^{2}}$$

Where e is the standard of error which was 0.05 (5%). Adjustment for non-response was done with an anticipated non-response rate of 15%. A total of 182 (100% response rate) mothers were interviewed in the two County Teaching and Referral Hospitals after proportionate allocation of the sample. To get the proportion to be interviewed from each site, sample size was divided by the population size then multiplied by the stratum size. The stratum size was represented by each facility where data collection was done. That is, n/N x stratum size (facility) where n = 182, N = 260, and the stratum size for KCTRH and VCTRH was 140 and 120 respectively. Therefore 98 and 84 respondents were interviewed from KCTRH and VCTRH respectively. The two County Teaching and Referral Hospitals were sampled purposively given the high numbers of preterm births registered in their District Health Information Systems (DHIS) report. Participants were sampled through simple random sampling with the NCU admission register being used as the sampling frame. Simple random sampling was done through lottery method where mothers from the admission register were given a number. After giving the mothers numbers, the researcher randomly drew numbers from the box to choose the sample. The process was applied in the two study sites. This was done to ensure that participants had an equal chance to participate in the study.

### Eligibility criteria

**Inclusion criteria:** mothers with preterm infants born in the hospital and consented to participate were included in the study.

**Exclusion criteria:** mothers with preterm infants with congenital abnormalities were excluded on assumption that they likely needed special attention given the state of their children. Those who were too sick to give consent were also excluded. Those whose infants had died during their stay in NCU were also excluded on the assumption that interviewing them could increase their emotional stress since they were grieving.

### Study variables

**Independent variable:** predictors were the independent variables and they included

demographic predictors, psychological, Psychiatry, environmental, immediate pregnancy and infant predictors.

**Dependent variable:** posttraumatic stress disorder score. That is, a respondent was categorised as having present or absent posttraumatic stress disorder using the impact of Event Scale –Revised (IES-R).

**Tools and procedures:** data were collected using data collection tools administered by the researcher and assisted by trained research assistants in the field of the study. The questionnaires were semi-structured and pretested and were used to obtain data on sociodemographic characteristics and predictors. Impact of event scale -revised (IES-R) was used to assess PTSD. This is a short self-report questionnaire of twenty-two questions. It is not a diagnostic tool for PTSD but measures the subjective response to a specific traumatic event, especially in the response set intrusion that is; nightmares, intrusive thoughts, imagery feelings and imagery, and dissociative-like experience and also measures avoidance and hyperarousal character of the mother [21]. A systematic review reported that impact of event scale questionnaire has high validity and reliability and is therefore beneficial for use in studies evaluating PTSD [22]. There are no cut-offs for the tool but scores below 24 means no PTSD while those exceeding 24 should be of great concern [21]. A score of 33 and above represents the best cut-off for a probable diagnosis of PTSD while a score of 37 and above (high PTSD) is high enough to suppress the immune system´s functioning. The tool did not require permission to use from the authors as long as they (the authors) are acknowledged.

**Data management and analysis:** on completion of data collection, data was cleaned, coded and entered into an Excel spreadsheet after which it was transferred into STATA version 15 for analysis. All the questionnaires were checked for missing data and all of them were complete. Descriptive statistics were analyzed in means and standard deviation (SD) for continuous variables and in percentages for categorical data with a significance level set at P ≤ 0.05. According to Gondwe [22], a score below 24 on the impact of event scale-revised (IES-R) was categorized as absent or no PTSD, 24 -32 was categorized as partial PTSD while a score of 33 was regarded as the cut-off for probable diagnosis of PTSD. Those mothers who had a score above 33 were categorized as having high PTSD. To determine the prevalence of PTSD therefore, all mothers who had a score above 24 were categorized as having PTSD. To determine the predictors associated with PTSD in NCU, logistic regression analysis was done where bivariate and multivariate analysis was carried out. Analyzed data was presented in frequency tables and texts.

**Ethical considerations:** approval to conduct the research was sought from Masinde Muliro University of Science and Technology (MMUST) Institutional Ethics Review Committee (IERC) after which research permit approval was given by the National Commission for Science, Technology and Innovation (NACOSTI). Permission was also sought from respective county governments and finally from Kakamega and Vihiga county referral hospitals´ administration. The purpose of the study was explained to the participants before seeking written informed consent from them. Participation was voluntary and respondents were liberal to withdraw at any stage of the study. Confidentiality was ensured by not having any sort of identification on the data collection tools. All the questionnaires collected were stored in lockable cabinets accessible only to the researcher and research team. Further, a password was used to protect electronic data in the computer.

## Results

**Descriptive statistics of sociodemographic characteristics for the mothers with infants hospitalized in neonatal care unit:** Table 1 shows the background characteristics of the respondents. One hundred and eighty-two (182) respondents recruited in to the study were interviewed. The majority of the respondents 67 (36.8%) were aged between 18-22 years and minority 34 (18.7%) were above 34 years. The majority 133 (73.1%) of the mothers in the study were married with 49 (26.9%) of them being single. Approximately 112 (84.2%) of the respondents within the study were from monogamous marriage with only 21 (15.8%) coming from polygamous family. Majority of the respondents 152 (83.5%) were christians while 29 (15.9 %) were Muslims. In terms of educational achievements, the majority of the respondents had attained secondary and tertiary level education at 86 (47.3%) and 51 (28.0%) respectively. Self-employment 41 (22.5%) and housewife 48 (26.4%) were the predominant occupation among the mothers.

**Table 1:** descriptive statistics of sociodemographic characteristics for the respondents

Socio-demographic variables	N= 182	%
**Age in years**		
18 - 22	67	36.8
23 - 27	41	22.5
28 - 33	40	22.0
Above 34	34	18.7
**Marital status**		
Single	49	26.9
Married	133	73.1
**Marriage type**		
Monogamous	112	84.2
Polygamous	21	15.8
**Religion**		
Christian	152	83.5
Muslim	29	15.9
Other	1	0.6
**Education**		
None	4	2.2
Primary	41	22.5
Secondary	86	47.3
Tertiary	51	28.0
**Occupation**		
Self - employment	41	22.5
Casual	39	21.4
White Collar	22	12.1
Housewife	48	26.4
Student	32	17.6

**Prevalence of post-traumatic stress disorder among respondents:** the prevalence of post-traumatic stress disorder was 78.6% (n=143) at a confidence interval of 95% CI: 0.72-0.84, cumulative of those with partial, moderate, and high post-traumatic stress disorder (Table 2).

**Table 2:** prevalence of post-traumatic stress disorder among respondents

Prevalence of post-traumatic stress disorder	n =182	%	
No post-traumatic stress disorder	39	21.4	
Partial Post-traumatic stress disorder	22	12.1	
Post-traumatic stress disorder	21	11.5	
High post-traumatic stress disorder	100	55.0	
**Outcome variable**	**n**	**%**	**95% CI**
No post-traumatic stress disorder	39	21.4	0.16-028
Present post-traumatic stress disorder	143	78.6	0.72-0.84

**Predictors associated with post-traumatic stress disorder among respondents in Western Kenya:** respondents who had previous preterm delivery were 0.09 less likely to develop post-traumatic stress disorder {AOR=0.09, 95% CI: 0.01-0.80, p=0.023}. Mothers who went through cesarean delivery were 11.1 times more likely to experience post-traumatic stress disorder {AOR=11.1, 95% CI: 1.1-114.8, p=0.043} while mothers living in fair housing conditions were 16.5 times more likely to report post-traumatic stress disorder {AOR=16.5, 95% CI: 1.7-156.2, p=0.014}. A number of the independent variables which were statistically significant at bivariate analysis but not significant at multivariate analysis include occupation, birth complication, counselling, time of counseling, support from the family, support from others and amount of support received (Table 3). However, neither age, marital status, type of marriage and religion were significant at the bivariate analysis.

**Table 3:** predictors associated with post-traumatic stress disorder among respondents in Western Kenya

Study variables (n=182)	No posttraumatic stress disorder	Post-traumatic stress disorder	COR	C.I	P-value	AOR	C.I	P-value
**Age in years**								
18-22	13 (33.3)	54 (37.7)	1.3	0.4-3.5	0.630	-	-	-
23-27	9 (23.1)	32 (22.4)	1.1	0.4-3.2	0.871	-	-	-
28-33	9 (23.1)	31 (21.7)	1.1	0.4-3.1	0.916	-	-	-
Above 34	8 (20.5)	26 (18.2)	ref	-	-	-	-	-
**Marital status**								
Married	10 (25.6)	39 (27.3)	1.1	0.5-2.4	0.839	-	-	-
Single	29 (74.4)	104 (72.7)	ref	-	-	-	-	-
**Marriage type**								
Monogamous	26 (89.7)	86 (82.7)	ref	-	-	-	-	-
Polygamous	3 (10.3)	1 (17.3)	1.8	0.5-6.6	0.369	-	-	-
**Religion**								
**Christian**	32 (82.1)	120 (83.9)	1.1	0.5-2.9	0.781	-	-	-
**Muslim**	7 (18.0)	23 (16.1)	ref	-	-	-	-	-
**Occupation**								
Self -employment	7 (18.0)	34 (23.8)	4.9	1.5-15.6	0.008	-	-	-
Casual	7 (18.0)	32 (22.4)	4.6	1.4-14.7	0.011	-	-	-
White collar	11 (28.2)	11 (7.7)	ref	-	-			
Housewife	6 (15.4)	42 (29.4)	7	2.1-23.1	0.001	-	-	-
Student	8 (20.5)	24 (16.8)	3	0.9-9.5	0.063	-	-	-
**Previous preterm delivery**								
Yes	12 (46.2)	17 (20.5)	0.3	0.1-0.8	0.012	0.09	0.01-0.80	0.023
No	14 (53.8)	66 (79.5)	ref	-	-	-	-	-
**Mode of delivery**								
Vaginal	37(94.9)	116 (81.1)	ref	-	-	-	-	-
Caesarean	2 (5.1)	27 (18.9)	4.3	1.0-19.0	0.054	11.1	1.1-114.8	0.043
**Birth complications**								
Birth traumas	1 (40.0)	12 (92.3)	18	1.2-271.5	0.037	-	-	-
Congenital abnormalities	3(60.0)	1 (7.7)	Ref	-	-	-	-	-
**Counselling on preterm**								
Yes	35 (89.7)	106 (74.1)	03	0.1-1.0	0.047	-	-	-
No	4 (10.3)	37 (25.9)	ref	-	-	-	-	-
**Time of counselling**								
Before delivery	14 (40.0)	22 (20.8)	ref	-	-	-	-	-
After delivery	21 (60.0)	84 (79.2)	2.5	1.1-5.8	0.026	-	-	-
**Family Support**								
Yes a lot	25 (64.1)	60 (42.0)	ref	-	-	-	-	-
Yes, a little bit	13 (33.3)	69 (48.3)	5.8	0.7-46.8	0.097	-	-	-
No not at all	1 (2.6)	14(9.7)	2.2	1.0-4.7	0.039	-	-	-
**Friends/ others support**								
Yes a lot	14 (35.9)	28 (19.6)	ref	-	-	-	-	-
Yes, a little bit	19 (48.0)	78 (54.5)	3.1	1.1-9.0	0.040	-	-	-
No, not at all	6 (15..4)	37 (25.9)	2.1	1.0-4.6	0.083	-	-	-
**Extent of support**								
Minimum	1(2.6)	23 (21.9)	18.6	2.3-152.3	0.006	-	-	-
Average	21 (53.8)	61 (58..1)	24	1.0-5.2	0.038	-	-	-
Maximum	17 (43.6)	21 (20.0)	ref	-	-	-	-	-
**Housing condition**								
Fair	14 (35.9)	80 (55.9)	2.6	1.2-5.4	0.011	16.5	1.7-156.2	0.014
Good	2	55 (38.5)	ref	-	-	-	-	-

* COR: crude odds ratio; AOR: adjusted odds ratio; CI: confidence interval; all the p<0.05 were considered statistically significant and bolded

**Impacts of event scale mean score:** the average score for intrusion score, avoidance score, and hyperarousal score was 1.69, 1.74, and 1.49 respectively. Most of the mothers as much as possible avoided situations that served as reminders of the trauma that they had experienced (mean =1.74) (Table 4).

**Table 4:** impact of events scale mean score

Subscales variable	Mean	Std
Intrusion score	1.69	0.80
Avoidance score	1.74	0.77
Hyperarousal	1.49	0.72

## Discussion

Maternal mental health outcomes vary widely in different countries and regions globally. This is often due to different instruments used in collecting data, the study design, sample sizes, the period of data collection, and the study population [23]. The prevalence of PTSD was 78.6% which is analogous to a prevalence of 77.8% from a systematic review on post-traumatic stress (PTS) symptoms following premature birth using the impact of event scale [21]. However, most of the studies utilized in the review were from high-income countries [18,21]. The similarity could have been due to the fact that the review presented a pooled prevalence. The prevalence of the present study is however high compared to the prevalence in other studies. A study carried out in the United States to determine the prevalence of maternal trauma 18 months after preterm birth noted a prevalence of 64.4% [24]. This prevalence is lower than that of this study and this could be attributed to the difference in the study population. During this study, data was collected from mothers whose infants were still in NCU against mothers 18 months after preterm birth. Malouf *et al*. [19] also noted in their review that the prevalence of PTSD declines over time and this might have led to the difference in the prevalence. The predictors reported during this study are not unique as a study carried out in Thailand on Factors Influencing Stress among mothers of preterm infants hospitalized in Neonatal Intensive care unit also reported that some of the predictors of stress included a cesarean section (delivery characteristics) and having an experience of previous preterm infant [24,25]. This is also supported by field [26] who in his review on postpartum anxiety prevalence, predictors and effects on child development also noted that having a cesarean delivery positively predicts mental health outcomes. This could be attributed to the fact that Thailand being a LMIC like Kenya tend to have almost similar challenges. Occupation, birth complication, counselling, time of counselling, support from the family, support from others and amount of support received weren´t statistically significant in multivariable analysis. This is contrary to other studies which indicated that these variables could influence PTSD scores [10,27]. A study in Iran by Naeem [10] to determine the incidence of post-traumatic stress disorder and associated risk factors among parents of hospitalized term and preterm neonates noted that the father´s occupation and mother´s employment status could positively predict PTSD. According to the study, mother´s employment may prevent the mother from fully taking charge of her baby in terms of helping in the care process and even limiting her (mother´s) time and energy to support the baby. On the father´s occupation, the same study noted that if the father is unemployed it could contribute more to stress-related economic problems leading to PTSD. In the current study, only mothers were interviewed and most of them were housewives. This could explain the difference in the study findings between the current study and that done in Iran.

**Implication to practice**: findings from the current study have significant implications to practice and highlight the need for routine mental health screening especially for PTSD for mothers with preterm babies in Neonatal care unit as part of standard care.

**Limitations:** being a cross-sectional study conducted in NCUs of county Teaching and Referral hospitals, the findings represent the population that had preterm infants in a specific NCU at a particular time of the year. Potential differences in practice may exist in private institutions and in National Teaching and Referral hospitals that may influence the generalizability of the findings, but this may be transferrable to similar settings. The study sample involved mothers and therefore the findings are likely to differ if fathers with preterm infants admitted to NCU were interviewed.

**Recommendations:** the study recommends that mothers with infants in NCU should receive assistance and information about their infants’ characteristics, treatment, behaviour and NCU environment before admission or during their stay in NCU as routine care in order to prevent PTSD. Special support and attention should also be given to those mothers with the reported predictors to prevent them from developing PTSD. Further research to determine the prevalence of PTSD among fathers and their associated factors should be carried out.

## Conclusion

The prevalence of PTSD was 78.6%. Although mothers of preterm infants experience stress, the associated predictors included caesarean section birth, and staying in fair housing conditions. In addition, some of the variables like occupation, social and family support, birth complications and counselling mothers before or after preterm birth were significant during the bivariate analysis but were not statistically significant during the multivariate analysis. This group of mothers may therefore require extensive support to prevent them from developing PTSD.

### What is known about this topic

- *Some of the independent predictors of post-traumatic stress disorder like previous exposure to trauma, occupation and social/family support have been studied elsewhere and are known*.

### What this study adds

- *This is the first study to determine the prevalence of post-traumatic stress disorder in Kenyan County Teaching and Referral Hospitals thus providing relevant evidence-based data which could be helpful in informing policy;*
- *The associated predictors in our setup are unknown*.
